# Analytical modelling of the transport in analog filamentary conductive-metal-oxide/HfO_x_ ReRAM devices†

**DOI:** 10.1039/d4nh00072b

**Published:** 2024-03-22

**Authors:** Donato Francesco Falcone, Stephan Menzel, Tommaso Stecconi, Matteo Galetta, Antonio La Porta, Bert Jan Offrein, Valeria Bragaglia

**Affiliations:** a IBM Research Europe – Zürich 8803 Rüschlikon Switzerland dof@zurich.ibm.com; b Peter Gruenberg Institute 7, Forschungszentrum Juelich GmbH 52425 Juelich Germany

## Abstract

The recent co-optimization of memristive technologies and programming algorithms enabled neural networks training with in-memory computing systems. In this context, novel analog filamentary conductive-metal-oxide (CMO)/HfO_x_ redox-based resistive switching memory (ReRAM) represents a key technology. Despite device performance enhancements reported in literature, the underlying mechanism behind resistive switching is not fully understood. This work presents the first physics-based analytical model of the current transport and of the resistive switching in these devices. As a case study, analog TaO_x_/HfO_x_ ReRAM devices are considered. The current transport is explained by a trap-to-trap tunneling process, and the resistive switching by a modulation of the defect density within the sub-band of the TaO_x_ that behaves as electric field and temperature confinement layer. The local temperature and electric field distributions are derived from the solution of the electric and heat transport equations in a 3D finite element ReRAM model. The intermediate resistive states are described as a gradual modulation of the TaO_x_ defect density, which results in a variation of its electrical conductivity. The drift-dynamics of ions during the resistive switching is analytically described, allowing the estimation of defect migration energies in the TaO_x_ layer. Moreover, the role of the electro-thermal properties of the CMO layer is unveiled. The proposed analytical model accurately describes the experimental switching characteristic of analog TaO_x_/HfO_x_ ReRAM devices, increasing the physical understanding and providing the equations necessary for circuit simulations incorporating this technology.

## Abstract 2

The recent co-optimization of memristive technologies and programming algorithms enabled neural networks training with in-memory computing systems.

## Graphical abstract

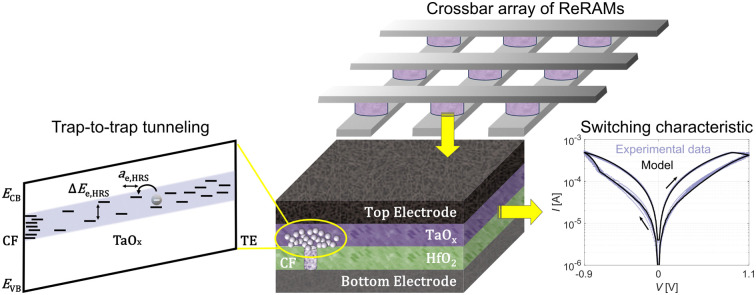

**New concepts**

Advancements in both technology and training algorithms are required to enable analog in-memory hardware execution of neural network training workloads. Due to the bidirectional accumulative analog response, conductive-metal-oxide (CMO)/HfO_x_ ReRAM devices may represent a technological breakthrough in this context. This work presents the first physics-based analytical model of the current transport and of the resistive switching in the CMO/HfO_x_ ReRAM technology. The experimental switching characteristic of these devices is described by a trap-to-trap tunnelling process according to the Mott and Gurney analytically formulation. The resistive switching is dominated by the defect dynamics in the CMO layer rather than in the conductive filament as per conventional metal/HfO_x_/metal ReRAM technology. Compared to such conventional ReRAM technology, the bidirectional accumulative analog response of the CMO/HfO_x_ ReRAM devices is attributed to the change from a full defect depletion/accumulation of a tiny gap in the conductive filament to a partial defect modulation in a larger half spherical CMO volume. Furthermore, the CMO is unveiled to act as both a thermal and electric field confinement layer in the CMO/HfO_x_ ReRAM devices. This study sets the groundwork for further CMO/HfO_x_ ReRAM technology optimization and provides the transport equations necessary for circuit simulations incorporating this technology.

## Introduction

### Analog AI inference and training

The recurrent data transfer between memory and processing units is a key source of speed and energy inefficiency in the hardware implementation of artificial neural networks (ANNs).^1^ To address this challenge, modern digital AI accelerators enhance computational parallelism and bring memory closer to the processing units.^2^ However, due to the limited on-chip memory capacity and the increasing number of parameters in modern ANNs, communication with off-chip memory is still needed.^3,4^ To overcome this memory wall, a promising approach, known as analog in-memory computing (AIMC),^5^ consists of transitioning to a hybrid architecture where some of the arithmetic and logic operations can be performed at the location where the data is stored.^6^ Emerging resistive memory (memristive) technologies such as redox-based resistive switching memory (ReRAM),^7^ phase-change memory (PCM)^8^ and magnetoresistive random access memory (MRAM),^9^ are particularly well suited for the AIMC computing paradigm.^10^ By organizing memristive devices in a crossbar configuration, matrix-vector multiplications (MVMs) can be performed directly in-memory by exploiting the multi-level storage capability of the devices, combined with Ohms law and Kirchhoffs law, in O(1) time complexity.^11,12^ AIMC-based AI hardware accelerator exploration has been mainly confined to inference workloads,^13^ being the accuracy of conventional training algorithms, such as the stochastic gradient descent (SGD), strongly dependent on the switching characteristics of the memristive devices.^14^ In particular, the key device requirement is the linear and symmetric conductance update in response to positive or negative pulse stimuli. However, since memristive devices exhibit non-ideal characteristics,^15,16^ advancements in both technology and training algorithms are required to achieve a full AIMC training system for deep learning workloads. Recent studies have demonstrated that parallel updates of AIMC ReRAM-based architecture may achieve a significant improvement in both energy efficiency and speed during neural network training with respect to digital AI accelerators.^17,18^ To address this challenge, a new ANN training algorithm, known as Tiki-Taka,^14^ was recently developed and optimized for analog resistive device. The core advantage of Tiki-Taka is to limit the device symmetry requirements from the entire conductance (*G*) window to a local symmetry point, where the changes in *G* are equal in both upward and downward directions.^14^ The key device requirement for the Tiki-Taka algorithm is analog resistive switching in both directions using a stream of identical pulses. To achieve so, the introduction of a properly engineered conductive-metal-oxide (CMO) in the conventional HfO_x_-based ReRAM metal/insulator/metal (M/I/M) stack has been shown to lead to crucial device optimization.^19^ Such M/CMO/I/M ReRAM devices exhibit improved characteristics in terms of analog states, stochasticity, symmetry point and endurance, compared to conventional M/I/M technology,^18^ paving the way for AIMC training units using ReRAM crossbar arrays.

### Origin of resistive switching

The origin of the resistive switching process in filamentary M/I/M HfO_x_-based ReRAM device is commonly attributed to the migration of oxygen vacancies in an oxygen-deficient conductive filament (CF).^20^ Such migration results in a purely filamentary conduction in the low resistance state (LRS) and tunneling transport through a low oxygen vacancy concentration region, known as gap, in the high resistance state (HRS).^20^ In contrast, the underlying mechanism behind the resistive switching in analog M/CMO/I/M HfO_x_-based ReRAM device is not fully understood, despite its device performance improvements. A recent study^21^ focuses on the electro-thermal modelling of static current transport in both LRS and HRS within a similar M/CMO/I/M ReRAM structure. However, the analytical current transport description and the switching dynamics, both crucial for a compact model description and consequently circuit simulations, remain unknown.^22,23^

### Analog CMO/HfO_x_ ReRAM devices

**Fig. 1:**
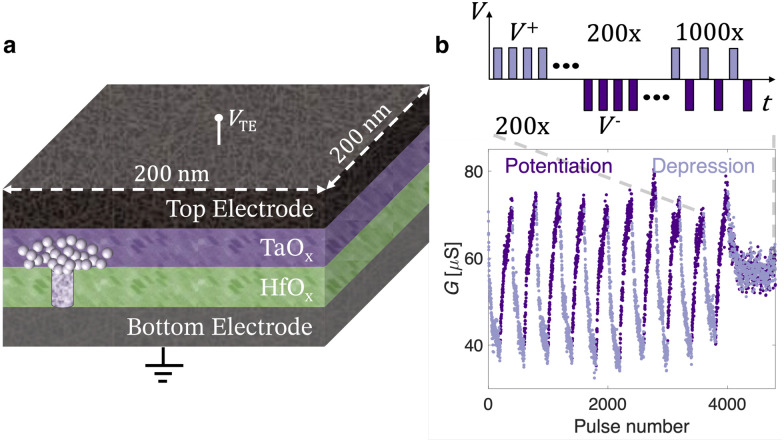
(a) Schematic illustration of the ReRAM device cross-section profile. (b) Bidirectional accumulative analog ReRAM response and symmetry point. The applied pulses have identical amplitudes of +1.6 V for the downward direction (*G* depression) and −1.4 V for the upward direction (*G* potentiation), each with a constant duration of 600 ns. After each pulse, the devices resistance is measured by 10 μs read pulses of ±0.2 V. The top inset shows the electrical testing scheme utilized to evaluate the analog switching properties of the ReRAM device for the Tiki-Taka algorithm.

**Fig. 2:**
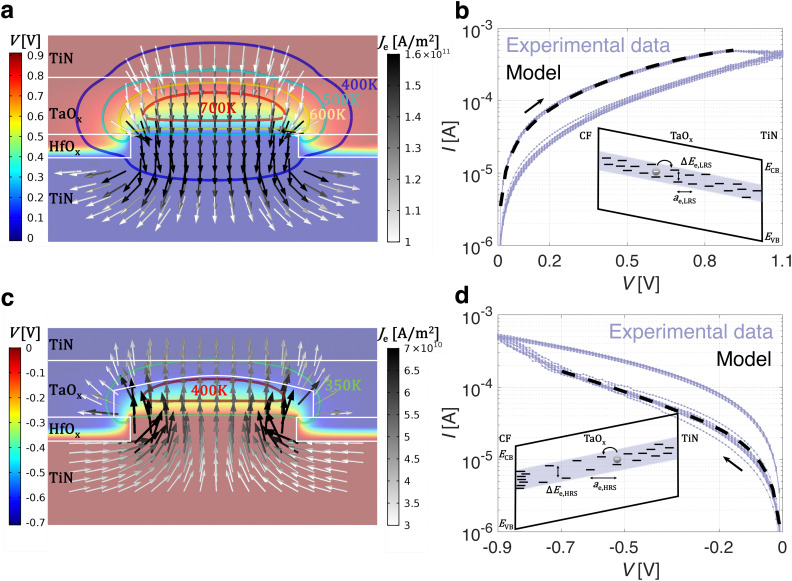
The steady-state 2D spatial distributions of *T*, *V* and *J*_e_ in a TaO_x_/HfO_x_ ReRAM device is shown (a) in LRS with a constant bias of 0.9 V and (c) in HRS with a constant bias of −0.7 V. Both *T* and *V* are locally confined in a 3D half-spherical volume in the TaO_x_ layer. The key purpose of the CF is the *J*_e_ confinement. The electro-thermal energy confinement in the TaO_x_ layer leads to the (a) RESET or (c) SET switching process. The experimental quasi-static *I*–*V* characteristic of a TaO_x_/HfO_x_ ReRAM device and the analytical electron trap-to-trap tunneling model are reported (b) in LRS and (d) HRS. The bottom insets in (b) and (d) show a schematic sketch of the trap-to-trap tunneling transport in the TaO_x_ sub-band, considering the CF and the TiN as electrodes, in LRS and HRS respectively.

This work addresses this challenge by providing a physics-based analytical model of the conduction in analog M/CMO/I/M HfO_x_-based ReRAM devices. The electron transport and the drift ion dynamics are both analytically described in LRS, HRS, intermediate analog states and during the SET (from HRS to LRS) and RESET (from LRS to HRS) transitions. The model results are compared to experimental data measured on a representative TiN/TaO_x_/HfO_x_/TiN ReRAM device, with a cell area of (200 nm)^2^ and the same stack as reported in our previous work.^24^Fig. 1a shows a simplified cross-sectional schematic of the analog TaO_x_/HfO_x_ ReRAM devices, in shared bottom electrode configuration, modelled in this work. The bidirectional accumulative ReRAM response under the application of trains of identical voltage pulses with alternating polarity in batches of 200, is illustrated in Fig. 1b. This pulse scheme is repeated 10 times, showing a stable *G* window. Finally, by alternately applying a single up pulse and down pulse, a stable *G* symmetry point is achieved. This process replicates the programming conditions utilized in Tiki-Taka training.^14^ As reported in our previous works,^25,26^ analog TaO_x_/HfO_x_ ReRAM devices are characterized by *I*_SET_ ≈ *I*_RESET_ and consequently *V*_SET_ ≈ *V*_RESET_. However, to increase the switching ratio (*R*_HRS_/*R*_LRS_), a larger stop voltage amplitude is desirable for the RESET compared to the SET process (|*V*_stop,RESET_| > |*V*_stop,SET_|). The fabrication process is detailed in our previous work,^24^ while additional information on the electrical characterization of ReRAM is reported in the Methods section of the ESI.† Although in Tiki-Taka training applications the targeted programming scheme employs short pulses (from hundreds of ps^27^ to μs^24^), quasi-static current–voltage (*I*–*V*) characterization of the ReRAM device is required to model the physics of the steady-state current transport. However, by cycling the TaO_x_/HfO_x_ devices with quasi-static voltage excitations, intentionally without current compliance control to prevent masking device physics during the switching, the conductance window shifts toward larger *G* values by a factor of around 3, as shown in Fig. 2b and d. Nevertheless, the physics-based analytical current transport model developed in this work and applied to such TaO_x_/HfO_x_ devices, can also be used to explain other similar analog M/CMO/I/M ReRAM systems, including those with lower average conductance.

## Physics-based analytical model

### Current transport: the model

In sub-stoichiometric metal-oxides, a significant concentration of defects related to oxygen vacancies and metal interstitials is observed.^28–30^ Each defect forms isolated donor-like states in the band gap of the metal-oxide, which become a sub-band for large defect concentrations.^28,31–33^ In oxides with a high band gap, there can be a substantial energetic separation (activation energy) between the defect states and the lower edge of the conduction band *E*_CB_. For example, in TaO_x_, the spectral location of the defect state sub-band associated with oxygen vacancies has been reported to be in the midgap, approximately 2 eV above the top of the valence band *E*_VB_.^32^ Consequently, according to the energy band alignment between the Fermi level of the electrodes and the defect sub-band in the conductive metal-oxide, it is likely that electrons coming from one electrode are trapped in the defect states during the current transport. However, to actively contribute to the conduction, trapped electrons must be released. Depending on the defect activation energy in the conductive metal-oxide, two scenarios may occur: the electrons get released by thermal excitation in the conduction band, or they tunnel from trap-to-trap until reaching the other electrode. In this work, due to midgap electronic states in the TaO_x_ layer, the electronic current *I*_e_ in a filamentary HfO_x_/TaO_x_ ReRAM device is modelled as a trap-to-trap tunneling process in the TaO_x_ layer, based on the description provided by Mott and Gurney.^34^ It describes electron-hopping conduction over an energy barrier Δ*E*_e_, which is identical in every direction in the absence of an electric field. Application of an electric field modifies the energy barrier by ∓*ea*_e_*E*/2 for forward (backward) jumps, resulting in a lowered (raised) barrier.1
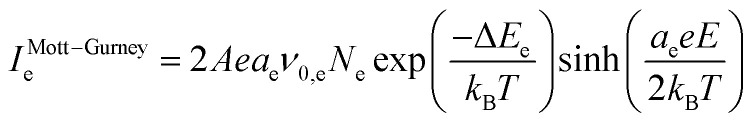


In eqn (1), *e* is the elementary charge, *k*_B_ is the Boltzmanns constant, *a*_e_ is the hopping distance, *ν*_0,e_ is the electron attempt frequency, *N*_e_ is the density of electronic defect states in the TaO_x_ band gap, Δ*E*_e_ is the zero-field hopping energy barrier, *T* and *E* are the local temperature and electric field, respectively, and *A* = π*r*_CF_^2^, *r*_CF_ being the filament radius, is the cross-sectional area of the filament at the interface with the TaO_x_ layer. Among the different types of defects in the TaO_x_ layer, this work focuses on oxygen vacancy defects 
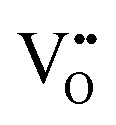
, which have been reported to generally have lower formation and migration energies with respect to Ta_i_ interstitials.^29,30^ Each double-positively charged vacancy 
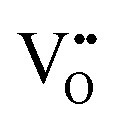
 generates two electronic defect states in the band gap.^30^ Therefore, the maximum density of defect states *N*^max^_e_ that may contribute to the transport can be related to the ionic vacancy concentration 
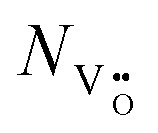
, according to 
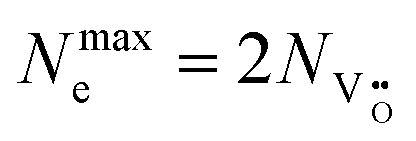
. Depending on the energy alignment between the defect sub-band in the TaO_x_ layer and the Fermi level of the electrodes, statistically only a sub-set of the defect states *N*_e_ contributes to the electronic transport, resulting in eqn (2).2
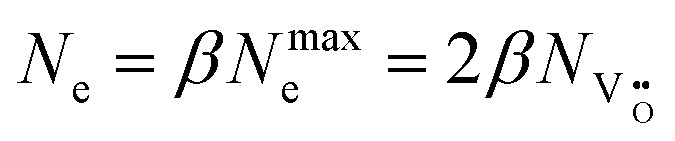
where *β* is a scaling factor between 0 and 1 to filter out the defect states that are not contributing to the conduction.

### Defect drift dynamics: the model

In addition to electronic transport, the description of the ion dynamics is crucial for understanding the resistive switching process and the resulting structural material changes. To do so, the drift dynamics of the ionic defects 
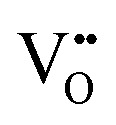
 in the TaO_x_ layer during the SET and RESET processes is similarly described by an ion-hopping conduction over a migration energy barrier Δ*E*_ion_,^35^ according to eqn (3) and (4),3
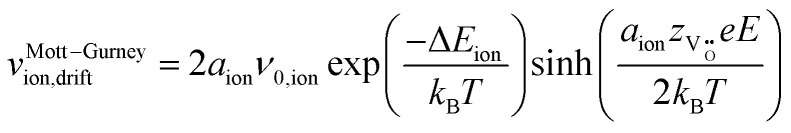
4
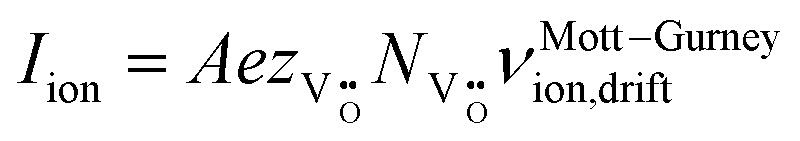
where *v*^Mott–Gurney^_ion,drift_ is the ion drift velocity, 
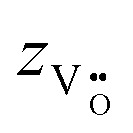
 is the charge number of the oxygen vacancies relative to the perfect crystal, *a*_ion_ is the crystal hopping distance and *ν*_0,ion_ is the ion attempt frequency.

### 3D finite element model (FEM)

The local temperature *T* and electric field *E* distributions in the steady state of TaO_x_/HfO_x_ ReRAM devices are assessed using a space resolved 3D FEM developed with COMSOL Multiphysics 5.2 software, according to eqn (5) and (6).

5 ∇·(*σ*(−∇*V*)) = 0

6 ∇·(−*k*∇*T*) = *J*_e_·*E* = *Q*_e_

where *J*_e_ is the electric current density, *σ* the electrical conductivity, *V* the electric potential, *k* the thermal conductivity and *Q*_e_ the heat source due to Joule heating. Table 1 lists the basic simulations parameters used in this work, taken from literature.

**Table 1:** Simulation parameters from literature

Symbol	Value	Symbol	Value
*ν*_0,ion_	4 × 10^12^ Hz^ ^^35^	*ν*_0,e_	2 × 10^13^ Hz^ ^^36^
*a*_ion_	0.4 nm ^37^	*k*_TaO_x__	1 W (m K)^−1^^38^
*k*_HfO_x__	0.5 W (m K)^−1^^39^	*k*_CF_	23 W (m K)^−1^^40^
*k*_TiN,Sputter_	3 W (m K)^−1^^41^	*k*_TiN,ALD_	11 W (m K)^−1^^42^
*σ*_HfO_x__	1 × 10^−4^ Sm^−1^	*σ*_CF_	4.2 × 10^4^ S m^−1^^21^
*σ*_TiN_	5 × 10^5^ S m^−1 ^^43^	Sweep rate	0.1 V s^−1^
*T*_0_	293 K	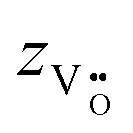	2

The boundary conditions include the current conservation to all the domains, and electric isolation (*n*·*J*_e_ = 0), where *n* is the outward normal vector, to all the vertical external boundaries such that no electric current flows through the boundaries. The electric potential is always applied to the ReRAM device top electrode, with the bottom electrode serving as the ground node, as shown in Fig. 1a. In the 3D FEM, all external boundaries of the ReRAM device are maintained at room temperature *T*_0_ in steady state, as negligible heating is expected far from the switching area. The model takes into account the entire experimental TaO_x_/HfO_x_ ReRAM device area of (200 nm)^2^.

## Results and discussion

### Analytical model of the LRS and HRS

An high-resolution scanning transmission electron microscopy (STEM) measurement on a similar TaO_x_/HfO_x_ ReRAM stack unveils a thin oxygen-rich TaO_x_ amorphous phase (≈3 nm) between the crystalline oxygen-deficient TaO_x_ and the HfO_x_ layer.^25,26^ The amorphous TaO_x_ phase, combined with the reduced oxygen scavenging capabilities of TaO_x_ material with respect to Ti,^44,45^ results in an increase of the forming voltage *V*_forming_ in the TaO_x_/HfO_x_ ReRAM device (≈3.6 V)^24^ compared to Ti/HfO_x_ technology (≈2.5 V).^26^ During the forming, a dielectric breakdown occurs in the HfO_x_ and oxygen-rich amorphous TaO_x_ layers, leading to the formation of a highly defect-rich conductive filament in both layers.^25^ For oxygen vacancy defects, reported formation energies range from 2.8 eV to 4.6 eV in HfO_x_, depending on the stoichiometry,^46,47^ and from 4.3 eV to 5.7 eV in TaO_x_, depending on the oxygen vacancy charge number.^48,49^ Due to the high formation energy, defect generation, both in HfO_x_ and TaO_x_ materials, occurs with statistical relevance only during the forming, when a large electric field and high temperature are locally reached. Therefore, the resistive switching process in a TaO_x_/HfO_x_ ReRAM device is determined by the migration of pre-existing defects, which leads to the modulation of the density of defect states in the band gap of the TaO_x_ layer. A 3D FEM is developed to simulate the electronic transport in a filamentary TaO_x_/HfO_x_ ReRAM device in both LRS and HRS. The electrical conductivity of the crystalline sputtered TaO_x_ layer is measured experimentally.^26^ However, due to the defect generation during the forming event, the local conductivity of the TaO_x_ layer on top of the conductive filament will be higher. For this reason, the electrical conductivity of the crystalline TaO_x_ layer was considered as a parameter in the 3D ReRAM model, within experimentally reported boundaries.^26^ From the model of the electronic conduction of the TaO_x_/HfO_x_ ReRAM device in the low-voltage linear regime in LRS, both the filament radius *r*_CF_ and the local conductivity of the TaO_x_ layer *σ*_TaO_x__ are extracted and reported in Table 2. Fig. 2a shows the steady-state 2D spatial distributions of temperature, electric potential, and current density in a TaO_x_/HfO_x_ ReRAM device in LRS with a constant bias of 0.9 V, taking into account the experimental *I*–*V* non-linearity with the same approach as reported in our previous work.^21^ The TaO_x_ layer, with its lower thermal conductivity (see Table 1), represents a larger thermal resistance compared to the conductive filament. For this reason, in steady state, a spatial temperature gradient exists within the TaO_x_ layer (see Fig. 2a), acting as a thermal confinement layer,^22^ confining heat on top of the conductive filament. In addition, due to the combination of the filamentary current constriction and the electrical conductivity of the TaO_x_ layer, the resistance in the LRS is dominated by the spreading resistance (*R*_spreading_ ∝ (2π*σ*_TaO_x__*r*_CF_)^−1^ ≫ *R*_CF_) between the CF and the TaO_x_, which leads to a nonuniform current density in the TaO_x_ layer.^21^ As a consequence, by properly designing its electrical conductivity,^21^ the TaO_x_ layer can also act as a field confinement layer, confining the applied electric field above the conductive filament in a half-spherical shape, as illustrated in Fig. 2a. Although the thermal and electric field confinement can similarly be achieved with an insulating metal-oxide layer, to avoid an increase of the overall forming voltage, a conductive TaO_x_ is used. The average temperature and electric field over such a 3D half-spherical volume in the TaO_x_ layer are calculated (see Fig. S1, ESI†), and used in eqn (1) to describe the static electronic conduction in LRS. Fig. 2b shows the experimental clockwise *I*–*V* quasi-static characteristic of a representative TaO_x_/HfO_x_ ReRAM device in LRS over 10 cycles. The analytical electron trap-to-trap tunneling model in the TaO_x_ layer (see Fig. 2b) accurately describes the experimental curve in LRS, enabling the extraction of *a*_e,LRS_, Δ*E*_e,LRS_, and *N*_e,LRS_, as reported in Table 2. Oxygen vacancy migration in the TaO_x_ layer has been reported as a process with an activation energy of ≈1.5 eV.^30,37^ As the applied positive electric potential on the TaO_x_/HfO_x_ ReRAM device increases, the electro-thermal energy in the TaO_x_ layer rises, through the local confinement of both the electric field and temperature. When the oxygen vacancy migration energy in the TaO_x_ layer is reached, the RESET switching process starts. During the RESET process, oxygen vacancies in the TaO_x_ layer radially migrate toward the conductive filament. Depending on the energy barrier at the TaO_x_/HfO_x_ interface, two scenarios may occur: an exchange of oxygen ions between the two layers, or an accumulation of oxygen vacancies within the TaO_x_ layer at the interface with the conductive filament.^50^ Both scenarios lead to a high resistance state (HRS) in the TaO_x_/HfO_x_ ReRAM device, characterized by a partial defect depletion of a half-spherical volume, also referred to as a dome,^21^ in the TaO_x_ layer. In this work, although it's not a binding assumption for the validity of the model, the resistive change of the TaO_x_/HfO_x_ ReRAM device is primarily attributed to oxygen vacancy migration within the TaO_x_ layer. In agreement with this interpretation, a 3D TaO_x,Dome_ volume (*V*_TaO_x_,Dome_), characterized by a lower density of defect states and therefore lower electrical conductivity *σ*_TaO_x_,Dome_ (see Table 2), has been employed to model electronic conduction in TaO_x_/HfO_x_ ReRAM devices in HRS. Fig. 2c shows the steady-state 2D spatial distributions of temperature, electric potential, and current density in a TaO_x_/HfO_x_ ReRAM device in HRS with a constant bias of −0.7 V, taking into account the experimental *I*–*V* non-linearity with the same approach as reported in our previous work.^21^ Similar to the LRS, TaO_x_ acts as both a thermal and field confinement layer (see Fig. 2c). The average temperature and electric field (see Fig. S1, ESI†) serve as inputs in eqn (1) to model the static current transport in HRS. The same analytical trap-to-trap tunneling model accurately describes the experimental curve of a TaO_x_/HfO_x_ ReRAM device also in HRS (see Fig. 2d). The physical parameters characterizing the trap-to-trap tunneling process in the TaO_x,Dome_, *a*_e,HRS_, Δ*E*_e,HRS_, and *N*_e,HRS_ are reported in Table 2. Compared to the LRS, the less-defective TaO_x,Dome_ volume is characterized by both a larger hopping distance *a*_e_ and energy barrier Δ*E*_e_, and a lower density of defect states *N*_e_ in the band gap. By increasing the applied negative electric potential, when the required activation energy is reached, the SET switching process starts. Oxygen vacancies accumulated in the TaO_x_ layer at the interface with the conductive filament, radially migrate back and repopulate the half-spherical TaO_x,Dome_, resulting in the LRS of the TaO_x_/HfO_x_ ReRAM device.

**Table 2:** Simulation results from our model

Symbol	Value	Symbol	Value
*r*_CF_	25 nm	*V*_TaO_x_,Dome_	3 × 10^−23^ m^3^
*σ*_TaO_x__	2 × 10^3^ Sm^−1^	*σ*_TaO_x_,Dome_	0.38 × 10^3^ Sm^−1^
*a*_e,LRS_	0.75 nm	*a*_e,HRS_	1.1 nm
Δ*E*_e,LRS_	55 meV	Δ*E*_e,HRS_	88 meV
*N*_e,LRS_	3.6 × 10^26^ m^−3^	*N*_e,HRS_	1.7 × 10^26^ m^−3^

### Intermediate resistive states

**Fig. 3:**
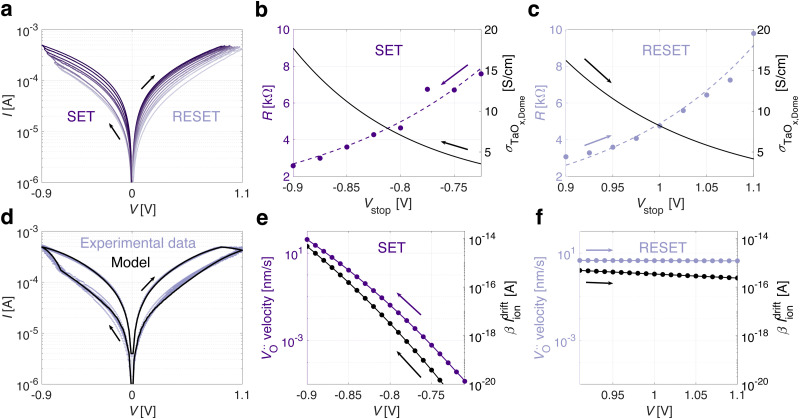
(a) Experimental quasi-static voltage sweeps, where the absolute stop voltage amplitude for both SET (from −0.725 V to −0.9 V) and RESET (from 0.9 V to 1.1 V) processes increases gradually with a step of 0.25 V. The exponential stop voltage-dependent evolution of the experimental intermediate resistive states measured at 0.2 V and the simulated equivalent electrical conductivity *σ*_TaO_x_,Dome_ are reported for SET (b) and RESET (c). (d) The analytical trap-to-trap tunneling model accurately describes the experimental switching characteristic of a TaO_x_/HfO_x_ ReRAM device. (e) Oxygen vacancy drift velocity and the resulting ionic current scaled by the *β* factor, are reported as a function of the applied quasi-static voltage for SET (e) and RESET (f), respectively.

By applying bipolar short pulse streams, the conductance of TaO_x_/HfO_x_ ReRAM devices can be gradually potentiated or depressed, as shown in Fig. 1b. The number of states and the overall analog switching performance have been shown to improve upon the application of ultrafast pulses (down to 300 ps).^27^ Additionally, in our previous work,^24^ it was demonstrated that TaO_x_/HfO_x_ ReRAM devices preserve analog properties after more than 10^7^ programming pulses, and show stable intermediate resistive states (<4% drift after 72 h at 85 °C). Due to the excellent endurance and retention properties of TaO_x_/HfO_x_ ReRAM devices, in first approximation, device non-idealities are not included in this work. The 3D FEM of the TaO_x_/HfO_x_ ReRAM device was used to model the intermediate resistive states resulting from the application of quasi-static excitations with increasing amplitudes. Fig. 3a shows the experimental response of a representative TaO_x_/HfO_x_ ReRAM device to quasi-static voltage sweeps with an increasing stop voltage amplitude *V*_stop_. Even with quasi-static excitations, intermediate resistive states can be accessed during both the SET and the RESET, without any external compliance current control. Fig. 3b and c illustrate a multistate exponential relationship between the experimental resistance measured at 0.2 V and *V*_stop_ during the SET and RESET processes, respectively. A comparable trend during the RESET of a filamentary ReRAM device has already been reported,^36^ but not for the SET process, which is typically a self-accelerated abrupt process.^20^ In agreement with the interpretation provided in this work (see Fig. 2), the equivalent electrical conductivity *σ*_TaO_x_,Dome_, assumed of a constant volume *V*_TaO_x_,Dome_, to model the electronic conduction in the low-voltage linear regime (see Fig. S2, ESI†), is extracted. Fig. 3b and c show the exponential evolution of the *σ*_TaO_x_,Dome_ as a function of *V*_stop_ during the SET and RESET processes, respectively.

### Analytical model of the switching characteristic

To model the current during the entire switching characteristic, in addition to the LRS and HRS (see Fig. 2), the transport during the SET and RESET transitions has to be addressed. To do so, the analytical trends of the electro-thermal conditions and trap-to-trap tunneling parameters during such transitions, are required. In Fig. 3b and c, both resistance, based on experimental evidence, and *σ*_TaO_x_,Dome_, based on model evidence, exhibit an exponential dependence on *V*_stop_ in the TaO_x_/HfO_x_ ReRAM devices. Additionally, the electro-thermal conditions (see Fig. S1, ESI†) and trap-to-trap tunneling parameters (see *a*_e_, Δ*E*_e_ and *N*_e_ from Table 2) are known at the onset of both the SET and RESET processes from the modelling of the static electronic transport. By combining these evidences, an exponential distribution for *T*, *E*, *a*_e_, Δ*E*_e_, and *N*_e_ within the static boundaries (from Fig. 2 and Table 2) is assumed to model the electronic current during both the SET and RESET transitions. Fig. 3d shows the resulting electron trap-to-trap tunneling transport model during the SET and RESET transitions according to eqn (1). The model in Fig. 3d accurately describes the experimental quasi-static switching characteristic, validating the previous assumption. During the RESET transition, the temperature slightly decreases while the electric field in the TaO_x_ layer increases (see Fig. S3, ESI†). The simulation aligns with the interpretation presented in this work, indicating an overall increase in resistance, resulting in reduced Joule heating and a progressively increasing electric field confined within the less-defective TaO_x,Dome_. During the SET transition, although the applied voltage increases, the electric field in the TaO_x_ layer stays nearly the same, while the temperature increases exponentially (see Fig. S3, ESI†). This aligns with a decrease in resistance, resulting in enhanced Joule heating and a defect-enrichment of the TaO_x,Dome_, restoring the LRS condition.

### 
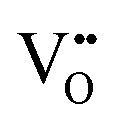
 drift dynamics

To explain the dynamics of the resistive switching in TaO_x_/HfO_x_ ReRAM devices, the drift of 
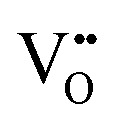
 during the SET and RESET transitions is modelled. For each voltage step (10 mV) during the SET and RESET processes, the average oxygen vacancy drift velocity is computed according to eqn (3). By scaling the drift velocity with the experimental measurement time (100 ms per step), an average drift induced displacement is computed for each voltage step. The cumulative sum of the displacements during both the SET and RESET transitions, results in the average size of the dome along the vertical direction. For consistency with the current transport model (see Fig. 2), an overall oxygen vacancy displacement of approximately 10 nm is assumed for both the SET and RESET, while the ion-hopping energy barriers Δ*E*_ion,SET_ and Δ*E*_ion,RESET_ are considered as fitting parameters. The extracted ion activation energies are Δ*E*_ion,RESET_ = 1.44 eV and Δ*E*_ion,SET_ = 1.32 eV. Consistently, both energies closely align with the reported oxygen vacancy migration energy of ≈1.5 eV^30^ in the TaO_x_ layer. The difference between Δ*E*_ion,RESET_ and Δ*E*_ion,SET_ may suggest that the two migrations happen through different energy paths. Oxygen vacancy accumulation at the interface with the conductive filament (during RESET) may require more energy than redistribution back into the 3D half-spherical volume (during SET). Fig. 3e and f show the oxygen vacancy drift velocity and the resulting ionic current scaled by the *β* factor, according to eqn (4), during the quasi-static SET and RESET, respectively. Although the *β* factor, which describes the sub-set of defect states contributing to conduction, may vary during the switching process, the change in *βI*^drift^_ion_ is primarily attributed to a change in *I*^drift^_ion_, which depends on the electro-thermal conditions according to eqn (4). In agreement with the experimental switching characteristic, the ionic drift dynamics follow an exponentially increasing trend during SET (see Fig. 3e) and a nearly uniform one during RESET (see Fig. 3f). Future work will focus on establishing the analytical link between the 
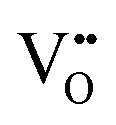
 average drift velocity and the ionic vacancy concentration 
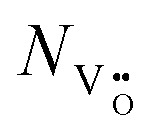
, which determines the electronic current according to eqn (1) and (2). Although not included in this work, the competing ionic diffusion and the reaching of a saturation of the oxygen vacancy concentration in the TaO_x_ layer, are expected to mitigate and eventually stop the drift dynamics. Compared to conventional Ti/HfO_x_ ReRAM technology, the proposed model of a TaO_x_/HfO_x_ ReRAM device shows a similar intrinsically gradual RESET, but a fundamentally different SET, from abrupt to exponential. The graduality of the SET process is explained by a new physical interpretation, from a full defect depletion/accumulation of a tiny gap in the HfO_x_ filament to a partial modulation of defects in a larger 3D half-spherical shape in the TaO_x_ layer. Additionally, the narrower resistive window (typically <10)^24^ in TaO_x_/HfO_x_ ReRAM devices yields a decreased temperature gradient during SET, enhancing its graduality.

### The role of the CMO layer: *σ*_CMO_ and *k*_CMO_

**Fig. 4:**
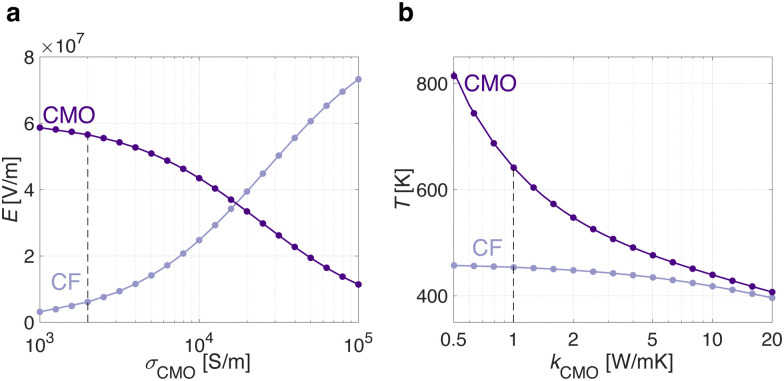
Considering a filamentary CMO/HfO_x_ ReRAM device in LRS with a constant applied bias of *V* = 0.9 V, the role of the electro-thermal properties of a designable CMO material is illustrated. By increasing the CMO electrical and thermal conductivity toward values typical of a metal, the spreading resistance and the resulting effectiveness of the electric field and temperature confinement in the CMO layer, decreases. The dashed lines indicate the values used in this work for the TaO_x_ material.

The proposed physical model is applicable to a range of analog bilayer ReRAM systems, including but not limited to TaO_x_/HfO_x_ ReRAM devices. Similarly to TaO_x_, other CMOs can be deposited in stable sub-stoichiometric phases.^21^ By replacing TaO_x_ with a designable CMO, the impact of its electrical *σ*_CMO_ and thermal *k*_CMO_ conductivities on the average electric field and temperature distributions is assessed in CMO/HfO_x_ ReRAM systems. To achieve this, the filament radius reported in Table 2 is used, along with a constant applied bias of *V* = 0.9 V on the top electrode of the CMO/HfO_x_ ReRAM device, assumed in LRS. Considering an arbitrary constant *k*_CMO_ = 1 W (m K)^−1^, like the one used for the TaO_x_ layer, Fig. 4a illustrates how the effectiveness of field confinement in the CMO layer (where *E*_CMO_ ≫ *E*_CF_) diminishes with the increase in CMO electrical conductivity *σ*_CMO_. Similarly, considering a fixed *σ*_CMO_ = 2 × 10^3^ Sm^−1^, like the one used for the TaO_x_ layer, Fig. 4b shows the thermal confinement in the CMO layer as a function of its thermal conductivity *k*_CMO_. For metallic metal-oxide, field confinement occurs within the conductive filament (*E*_CF_ ≫ *E*_CMO_) and the temperature is equally distributed between the CMO and the CF, leading to the conventional abrupt switching process dominated by the CF in Ti/HfO_x_ ReRAM device. On the other hand, properly designing the CMO as a thermal and electric field confinement layer is expected to yield an analog bilayer ReRAM device according to the physical model described in this work.

## Conclusions

Advancing the physical understanding of the resistive switching process in analog CMO/HfO_x_ ReRAM devices is fundamental to further improving the hardware performance and enabling on-chip training of large neural network models with this technology. In this work, the first physics-based analytical current transport model of analog CMO/HfO_x_ ReRAM devices is presented. As a case study, the model is employed to simulate experimental data from a representative analog TaO_x_/HfO_x_ ReRAM device. Table 3 presents a comparison of this work with available ReRAM models in literature. Based on this work, future studies will focus on the development of a compact model description including device failure mechanisms, and subsequently circuit simulations of analog CMO/HfO_x_ ReRAM technology.

**Table 3:** Comparison with available ReRAM models in literature

	Bengel *et al.*^51^	Wu *et al.*^22^	Falcone *et al.*^21^	This work
	Jiang *et al.*^52^			
Device	ReRAM	ReRAM	ReRAM	ReRAM
Materials	HfO_x_	TaO_x_/HfO_x_	CMO/HfO_x_	TaO_x_/HfO_x_
Synapse	Digital	Analog	Analog	Analog
Model	Analytical	Empirical	Empirical	Analytical
Transport	*e* and 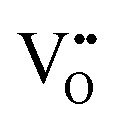	*e*	*e*	*e* and 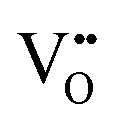
Approx.	Compact model	FEM	FEM	FEM

In this work, it is shown that the combination of the filamentary current constriction with the electro-thermal properties of the TaO_x_ material, enables temperature and electric field confinement in the TaO_x_ layer. Consequently, the resistive switching is dominated by the defect dynamics in the TaO_x_ layer rather than in the conductive filament as per conventional M/I/M ReRAM stack. The proposed analytical current transport model describes the Mott–Gurney trap-to-trap tunneling process. The resistive switching of the analog ReRAM device is attributed to the modulation of the density of defect states in the sub-band of the TaO_x_ layer. During the RESET switching process, oxygen vacancies in the TaO_x_ layer radially migrate toward the conductive filament, partially depleting a half-spherical volume, also known as dome. As a consequence, the lower density of defect states results in a lower electrical conductivity of the TaO_x,Dome_. In this process, the electric field increases while the temperature decreases. Conversely, the SET process, characterized by an exponentially increasing temperature and a nearly constant electric field, consists of a defect-enrichment of the TaO_x,Dome_, restoring the LRS condition. The more gradual SET of TaO_x_/HfO_x_ ReRAM devices compared to conventional Ti/HfO_x_ technology is attributed to the change from a full defect depletion/accumulation of a tiny gap in the conductive filament to a partial defect modulation in a larger TaO_x,Dome_. Additionally, the narrower resistive window in TaO_x_/HfO_x_ ReRAM device yields a decreased temperature gradient during the SET, enhancing its graduality. The intermediate resistive states are described as a gradual increase/decrease of the TaO_x,Dome_ defect density, which results in a variation of its electrical conductivity. The SET and RESET activation energies, extracted from the modelling of the oxygen vacancy drift dynamics, closely align with the reported oxygen vacancy migration energy in the TaO_x_ layer. The physics-based analytical current transport model developed in this work is not limited to TaO_x_/HfO_x_ devices, but applies to other similar analog CMO/HfO_x_ ReRAM systems. Eventually, the role of the *σ*_CMO_ and *k*_CMO_ on the electric field and temperature distributions is described, setting the groundwork for further CMO/HfO_x_ ReRAM device optimization.

## Author contributions

Conceptualization: D. F. F. and V. B.; methodology and validation: D. F. F., S. M. and V. B.; simulations: D. F. F.; result interpretation: D. F. F., S. M., M. G., T. S., V. B., A. L. P. and B. J. O.; data curation: D. F. F. and V. B.; hardware fabrication: T. S.; electrical characterization: D. F. F. and T. S.; supervision: V. B. and S. M.; manuscript writing: D. F. F.; manuscript review and editing: all authors; funding acquisition: B. J. O. and V. B.

## Conflicts of interest

There are no conflicts to declare.

## Supplementary Material

NH-009-D4NH00072B-s001
