# Supplementary material for: Analytical modelling of the transport in analog filamentary conductive-metal-oxide/HfOx ReRAM devices
Source: Nanoscale Horiz. 2024 Mar 22;9(5):775–84. doi: 10.1039/d4nh00072b (PMC11057356; doi:10.1039/d4nh00072b)
Supplement: NH-009-D4NH00072B-s001 [file NH-009-D4NH00072B-s001.pdf]

Electronic Supplementary Material (ESI) for Nanoscale Horizons.
This journal is © The Royal Society of Chemistry 2024

Electronic Supplementary information to manuscript:

"Analytical Modelling of the Transport in Analog Filamentary Conductive-Metal-Oxide/HfO$_x$ ReRAM Devices"

Donato Francesco Falcone,[a*] Stephan Menzel,[b] Tommaso Stecconi,[a] Matteo Galetta,[a] Antonio La Porta,[a] Bert Jan Offrein[a] and Valeria Bragaglia[a]

[a] IBM Research Europe - Zürich, 8803 Rüschlikon, Switzerland.
[b] Peter Gruenberg Institute 7, Forschungszentrum Juelich GmbH, 52425 Juelich, Germany.
[*] E-mail: dof@zurich.ibm.com

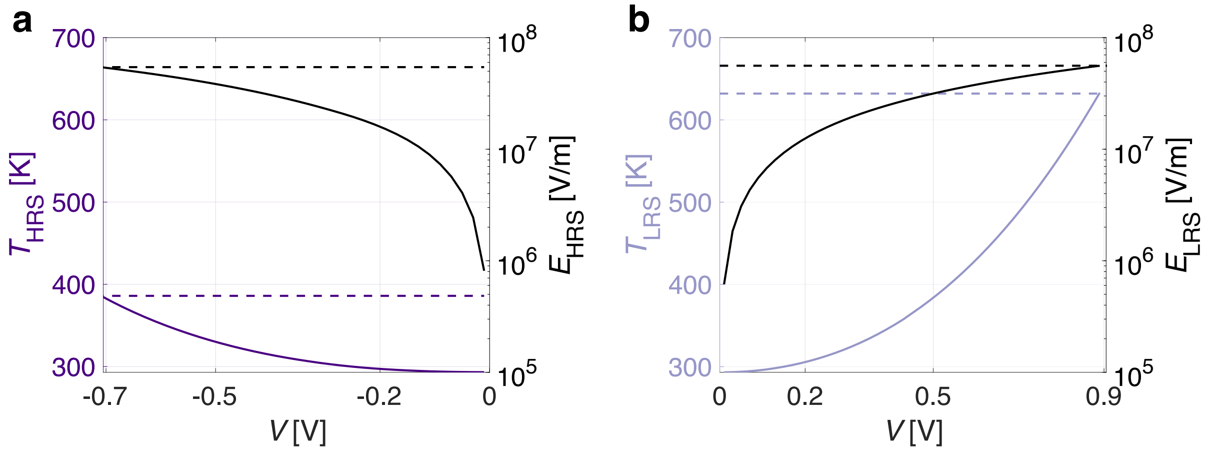

Figure S1: The voltage-dependent evolution of the average temperature and electric field over the 3D half-spherical volume ($V_{TaOx,Dome}$) in HRS (a) and LRS (b). The dashed lines represent the electro-thermal conditions at the onset of *SET* (a) and *RESET* (b) processes.

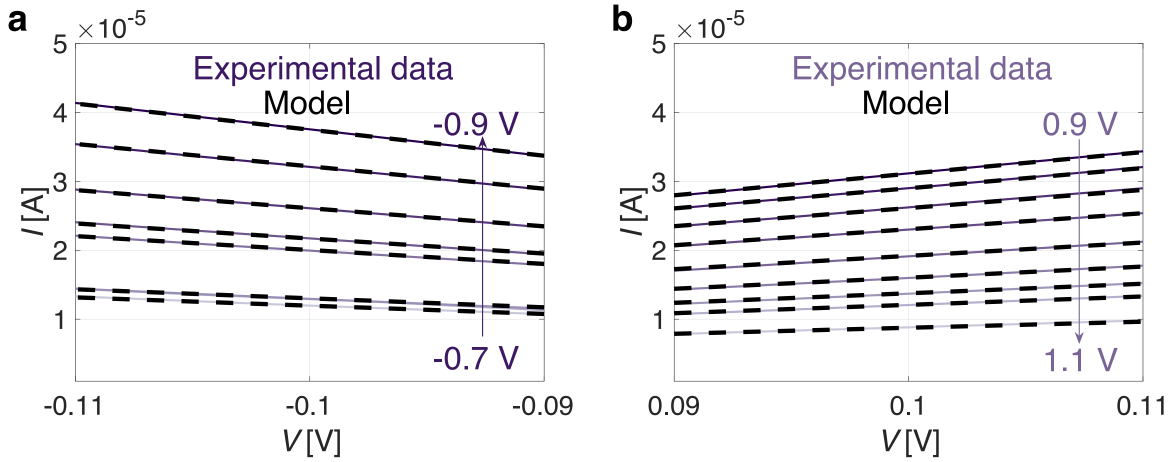

Figure S2: Experimental and simulated (dashed lines) intermediate resistive states due to quasi-static voltage sweeps, where the absolute stop voltage amplitude (V_{stop}) for both *SET* (a) and *RESET* (b) processes increases gradually, according to Figure 3a. By modelling the current of each intermediate state in the low-voltage (0.1 V) linear regime, the equivalent electrical conductivity $\sigma_{TaOx,Dome}$, assumed of a constant volume $V_{TaOx,Dome}$, is extracted and reported in Figures 3b and 3c, respectively. The choice of modelling the low-voltage regime allows to extract the equivalent intermediate $\sigma_{TaOx,Dome}$ where the device response is linear, hence temperature and electric field induced non-linearities are negligible.

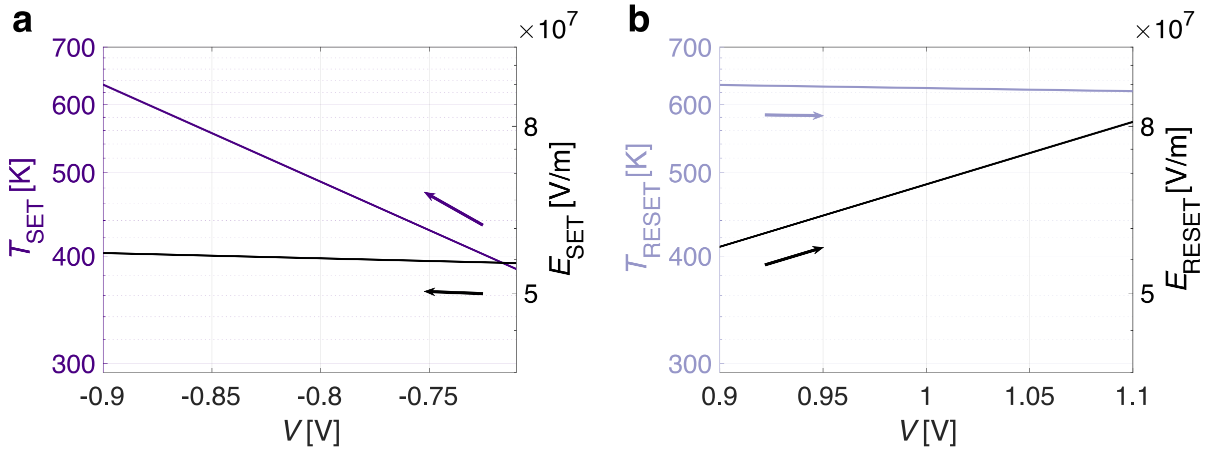

Figure S3: Assumed exponential trend of the temperature and electric field during both *SET* (a) and *RESET* (b) switching processes. Both exponential trends are validated by the accurate description provided by the analytical trap-to-trap model of the experimental switching characteristic (see Figure 3d). The electro-thermal condition at the onset of *SET* and *RESET* are provided in Figure S1. The electro-thermal endpoint of *SET* at -0.9 V corresponds to the onset of *RESET* at 0.9 V, while that of *RESET* at 1.1 V has been computed extending the 3D FEM of ReRAM device in HRS.

Methods

Electrical characterization

The pulsed electrical characterization is conducted using a NI PXIe-5451 arbitrary waveform generator to deliver the generated pulses to the device top electrode, and a NI PXIe-5164 oscilloscope to measure the current signal flowing through the bottom electrode. Figure 1b in the manuscript shows the programming pulse scheme. After each programming pulse, the device's resistance is measured by alternating positive and negative pulses with amplitudes of ±200 mV and a duration of 10 μs, to cancel out any potential measurement offset. The quasi-static current-voltage (I-V) characterization of the ReRAM devices is performed using an Agilent B1500A Semiconductor Device Parameter Analyzer. A constant voltage step of 10 mV and a measurement time of 100 ms/step, with a sweep rate of 0.1V/s, have been used during the quasi-static I-V characterization of the ReRAM devices.
